# Electrospun Functional Nanofiber Membrane for Antibiotic Removal in Water: Review

**DOI:** 10.3390/polym13020226

**Published:** 2021-01-11

**Authors:** Kun Zhao, Shi-Xiong Kang, Yao-Yao Yang, Deng-Guang Yu

**Affiliations:** School of Materials Science & Engineering, University of Shanghai for Science & Technology, 516 Jun-Gong Road, Shanghai 200093, China; 183762604@st.usst.edu.cn (K.Z.); 182442511@st.usst.edu.cn (S.-X.K.); yyyang@usst.edu.cn (Y.-Y.Y.)

**Keywords:** electrospinning, electrospun functional fiber membrane, antibiotics, adsorption, photocatalysis, biodegradation

## Abstract

As a new kind of water pollutant, antibiotics have encouraged researchers to develop new treatment technologies. Electrospun fiber membrane shows excellent benefits in antibiotic removal in water due to its advantages of large specific surface area, high porosity, good connectivity, easy surface modification and new functions. This review introduces the four aspects of electrospinning technology, namely, initial development history, working principle, influencing factors and process types. The preparation technologies of electrospun functional fiber membranes are then summarized. Finally, recent studies about antibiotic removal by electrospun functional fiber membrane are reviewed from three aspects, namely, adsorption, photocatalysis and biodegradation. Future research demand is also recommended.

## 1. Introduction

Water is one of the resources for human survival and is the most abundant covering 3/4 of the Earth’s surface [1]. However, the available freshwater accounts for approximately 3% of the total amount of water on the planet. Hence, water is a precious and scarce resource. Water pollution by the heavy industry, chemical industry and other highly polluting enterprises has dramatically aggravated the shortage of freshwater worldwide. The World Watch Institute stated that water shortages may affect more than 2/3 of the world’s population by 2025, including almost every country in the world, even developed ones [2]. Therefore, the treatment of sewage and wastewater has become a hot research topic.

Many kinds of pollutants, such as colored dyes [3,4,5], heavy metal ions [6,7,8], harmful bacteria [9,10,11] and drugs [12,13,14], are harmful to water. Among them, antibiotics have attracted attention as a new water pollutant [15,16]. These substances are compounds synthesized partly or entirely by microorganisms to kill or inhibit the growth of other organisms [17]. Antibiotics have many types. Table 1 classifies the common antibiotics according to their mechanism and includes statistics on some of their physical and chemical properties. In the early 20th century, the discovery of antibiotics has brought good news to human beings. This drug is widely used to treat human and animal diseases caused by microbial infections [18] and as a growth promoter in agriculture, aquaculture, beekeeping, livestock and other fields [19].

The antibiotics consumed by humans and animals cannot be fully absorbed by the body [20], and approximately 10–90% are discharged into the environment in the form of parent compounds or biodegradable metabolites [21]. Antibiotic pollutants are difficult to biodegrade and thus can exist stably for a long time in the environmental matrix [22]. In addition, antibiotic contaminants seriously influence antibiotic-resistant bacteria and genes, thus reducing the effectiveness of traditional antibiotics and promoting the production of drug-resistant bacteria [23]. These substances may also combine with other pollutants in water to produce highly toxic compound pollutants [24]. Antibiotics pose a potentially enormous threat to human health and ecosystems [25]. In 1982, Watts, C. D. [26] detected drugs (macrolides, sulfonamides and tetracyclines) in the river water—the first case of antibiotic contamination in surface water. Currently, the level of antibiotics reaches the range of ng/L in surface water, groundwater and seawater; its amount in low of urban sewage and generally high in the range of mg/L in hospital sewage [17]. Hence, antibiotic wastewater treatment is urgent.

**Figure 1:**
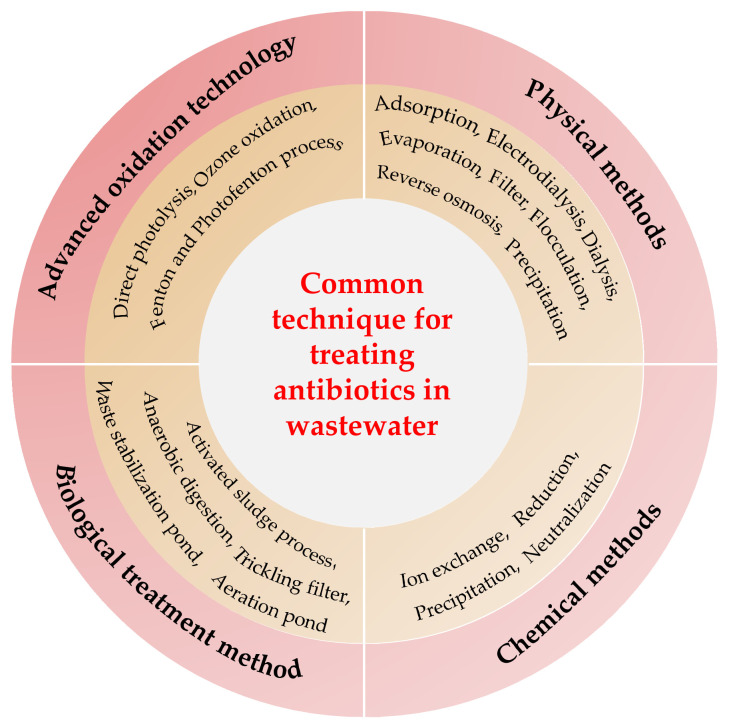
Techniques commonly used to treat antibiotics in wastewater.

The sewage treatment plant is the last barrier before the wastewater is discharged into the environment. Manvendra et al. [27] pointed out that the sewage treatment plant is designed to degrade the easily and moderately degradable organic matter within the range of mg/L. However, antibiotics mainly exist in in the water at the concentration of ng/L to μg/L and exhibit high activity. Therefore, the sewage treatment plant can partially remove antibiotics but is not suitable for their complete removal. Figure 1 summarizes the following existing techniques for the removal of antibiotic pollutants in water: (1) physical method, which changes the drug from the liquid phase to the solid phase; (2) chemical and biological treatments, which transform the drug into new metabolites and degradation products through chemical reactions [27] and (3) advanced oxidation (mainly ozonation, Fenton and Photo Fenton processes), which includes physical (such as physical adsorption) and chemical reactions. The application of these technologies has achieved good results in the treatment of antibiotic-contaminated wastewater. However, the process must be continuously updated.

**Figure 2:**
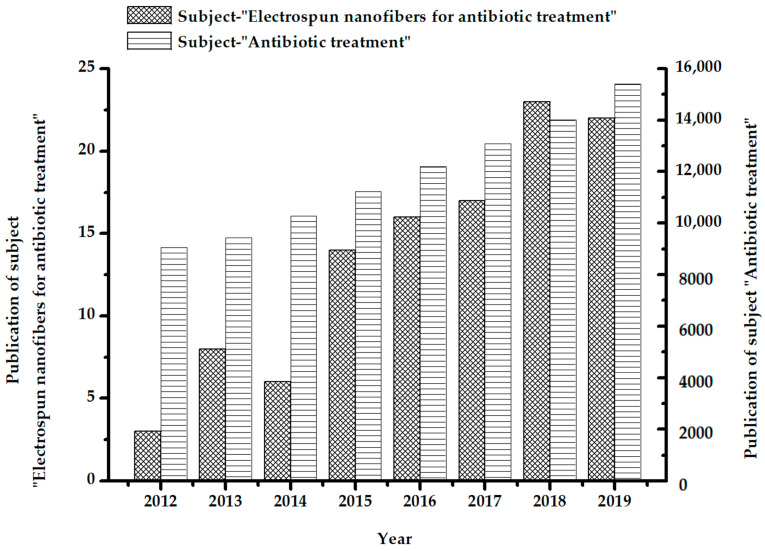
Statistics of literature retrieved on the “Web of Science” platform with the theme of “Electrospun nanofibers for antibiotic treatment” and “Antibiotic treatment”.

Electrospun fiber membrane has the advantages of a small diameter, large specific surface area, high porosity (up to 80% or higher), good connectivity, small pore size, high surface roughness and low gram weight [28]. The advantages of existing technologies to treat antibiotic wastewater can be fully utilized by using electrospun fiber membrane as a load functional substance carrier. Nanofiber-based composites can overcome inherent limitations of bare nanofiber systems (e.g., physical, chemical and catalytic properties) and remove antibiotic pollutants more effectively. Studies on the topics of “Electrospun nanofibers for antibiotic treatment” and “Antibiotic treatment” were searched on the “Web of Science” platform, and the results are shown in Figure 2. The number of related articles published on the two topics has generally increased every year. The total number of articles retrieved on the subject of “Antibiotic treatment” is more than 9000 articles per year from 2012 to 2019, indicating that the topic of antibiotic removal is highly relevant. However, the maximum number of articles corresponding to the theme of “Electrospun nanofibers for antibiotic treatment” is no more than 30 articles per year on the platform. This value indicates a room for improvement for the use of electrospun fiber membrane in antibiotic pollution control.

## 2. Electrospinning

In wastewater treatment, polymer membranes have shown excellent application potential due to their superior performance. To date, the preparation technologies for polymer membranes include electrospinning, template synthesis, interfacial polymerization, self-assembly, phase inversion, stretching, sintering and track etching [30,31]. As a “top-down” membrane technology [32], electrospinning can prepare fibers with a diameter in the range of several hundred nanometers in large quantities within a short time [33]. The diameter and orientation of the fibers can also be easily controlled [34]. This technology also has the advantages of simple operation, low cost, high efficiency and excellent flexibility. Therefore, electrospun fiber membranes have become research hotspots in the field of water treatment [35]. An in-depth research on electrospinning technology is warranted.

### 2.1. Initial Development History of Electrospinning

**Figure 3:**
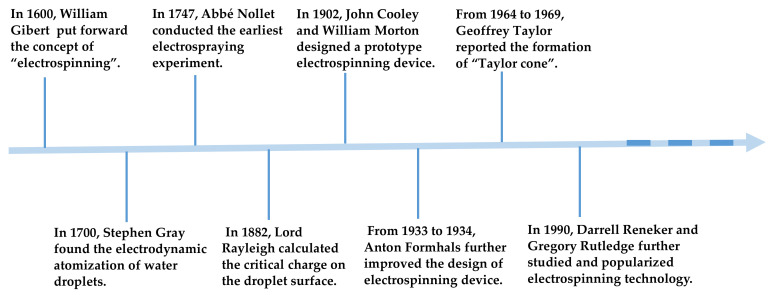
Initial development history of electrospinning technology.

The creation and development of each technology are accompanied by the tortuous process and grinding of time, and electrospinning is no exception. Figure 3 summarizes the primary development history nodes of electrospinning. As early as 1600 A.D. William Gibert [36] found that a droplet interacts with the electrostatic field force in a high-voltage electric field. When a certain amount of electric charge is present on its surface, the droplet deforms into a cone shape and a tiny droplet is ejected from the tip of the cone. He also put forward the concept of “electrospinning”. At the beginning of the 18th century, Stephen Gray mentioned that water droplets undergo electrodynamic atomization in the electrostatic field, and tiny jets are ejected from the tip of the liquid during this process [37]. In 1747, Abbe Nollet performed the earliest known electrospraying experiments and found that water could be sprayed into aerogel in an electrostatic field [38]. Since then, theoretical research on electrospinning has propagated. In 1882, Lord Rayleigh [39] theoretically estimated the maximum charge on the surface of droplet before straight jet injection and provided an essential theoretical basis for further research on the principle of electrospinning. In 1902, John Cooley [40] and William Morton [41] applied for two patents to design prototypes of an electrospinning device. From 1934 to 1944, Anton Formhals successively applied for some patents to improve the electrospinning device, thus allowing the electrospinning technology to move toward commercial production [42]. The preparation technology for electrospinning has matured with the continuous research efforts. However, the understanding of electrospinning mechanics is still in the early stages. In 1964–1969, Geoffrey Taylor published many pioneering works using mathematical language and modeling to describe the change process of solution or melt droplet from spherical to conical under the action of a high-voltage electric field [43,44,45]. Owing to his outstanding contribution, the cone-shaped hemisphere formed by droplets under high-voltage electric field is called “Taylor cone” [46]. In the next 20 years, electrospinning technology did not attract attention from the scientific research and industrial circles. In 1990, some research groups represented by Darrell Reneker and Gregory Rutledge reinvented and promoted electrospinning technology. Their works confirmed that a large number of polymer solutions could be electrospun into fiber membranes [47]. This discovery brought new life to electrospinning and has ushered it in a prosperous period of development. A search with the subject word “Electrospinning” was conducted on the “Web of Science” and produced 42,269 items (11 December 2020). This number will continue to increase in the near future.

### 2.2. Working Process and Principle of Electrospinning

**Figure 4:**
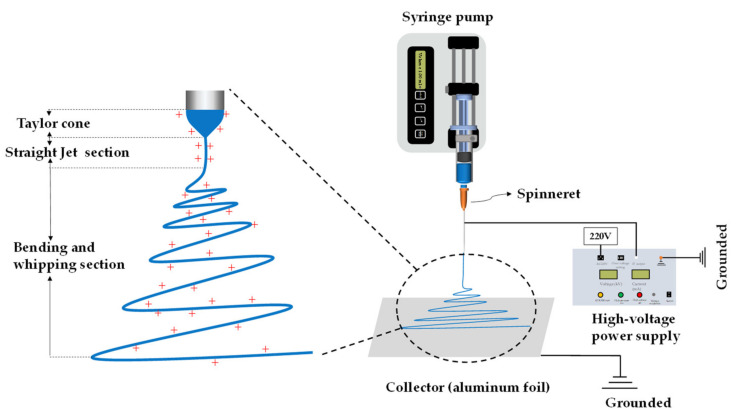
Schematic of the electrospinning process and apparatus.

Electrospinning is an advanced fiber preparation technology that uses the interaction between the liquid and electric field to generate products [48]. The apparatus and the process are shown in Figure 4. The electrospinning apparatus is mainly composed of the syringe pump, syringe, spinneret, high-voltage power supply and collector device (commonly used types are aluminum foil and roller). The process and technical principle of electrospinning can be divided into the following three stages [49]. (1) Driven by the syringe pump, the liquid inside the syringe is extruded from the spinneret. The droplet forms a spherical shape at the tip of the spinneret due to the balance of surface tension and gravity. (2) When the electric field force is applied, the surface morphology of the droplet gradually changes from spherical to a conical shape due to the enrichment of electric charges. This process is controlled by gravity, Coulomb force and surface tension. The tip of the cone emits straight jet when the Coulomb force is greater than the surface tension. (3) According to the Rayleigh instability principle and the interaction of the positive charges on the jet surface in the electric field, the duration of the straight jet section is short and thus results in bending and whipping. Owing to Coulomb repulsion force and gravity interaction on its surface, the jet is stretched rapidly in the bending whip section and then solidified into the fiber with the rapid solvent evaporation. The critical voltage of emitting straight jet at hemispherical cone tip can be calculated according to the formula [50]:

(1) $$V_{c}=\sqrt{\frac{\gamma d^{2}}{\varepsilon R}},$$

where $V_{c}$ is the critical voltage of emitting straight jet at the hemispherical cone tip (kV), γ is the surface tension of the hemispherical cone (kg/s^2^), d is the distance between the positive and negative electrodes (m), ε is the dielectric constant (C^2^/(Jm)) and R is the curvature (m) of the hemispherical cone. When the applied voltage $V_{c}$ exceeds the critical voltage, the charge on the hemispherical cone surface reaches the critical charge and the tip of the cone can emit straight jet to start the electrospinning process.

Electrospraying is also a common technology, and electrospinning is a special case of electrospraying [51,52,53]. Their main difference is the viscosity of working fluids that results in various jet behaviors. Electrospinning involves highly viscous working liquid, and the jet can maintain a continuous form to produce fibers. Electrospraying involves low-viscosity working liquid, and the jet cannot maintain a consistent form and splits into particles [28]. The entanglement number ${\left(n_{e}\right)}_{soln}$ can be used to quantitatively calculate whether the polymer solution can be spinnable as shown in the following formula [54]:

(2) $${\left(n_{e}\right)}_{soln}=\frac{M_{W}}{{\left(M_{e}\right)}_{soln}}=\frac{\varphi M_{W}}{\left(M_{e}\right),}$$

where $M_{W}$ is the relative molecular weight of the polymer, $M_{e}$ is the entangled molecular weight that mainly refers to the average molecular weight between entangled knot and φ is the polymer concentration. When ${\left(n_{e}\right)}_{soln}$ < 2, nanoparticles are produced by pure electrospraying. When 2 < ${\left(n_{e}\right)}_{soln}$ < 3.5, the nanoparticles gradually change into bead-like fibers (or spindles-like fibers). When ${\left(n_{e}\right)}_{soln}$ > 3.5, continuous fibers are produced by pure electrospinning due to the sufficient chain entanglement of the polymer solution.

### 2.3. Influencing Factors of Electrospinning

The parameters affecting the morphology of electrospun nanofibers can be grouped into three categories: system, process and environmental [55]. System parameters include polymer molecular weight, polymer solution concentration, polymer solution conductivity, dielectric constant, surface tension, viscosity and solvent type; process parameters mainly refer to the applied voltage, fluid flow rate, linear receiving distance from the tip of the spinneret to the collector and diameter of spinneret; and environmental parameters mainly include the humidity and temperature of the surrounding environment [56]. The influence of these parameters on the morphology of the electrospun fibers must be within a specific range, and the interaction between the parameters should be considered. Table 2 summarizes the effects of these parameters on the morphology of electrospun nanofibers.

#### 2.3.1. System Parameters

System parameters are mainly related to the properties of polymer solution for electrospinning and mainly affect the morphology of electrospun nanofibers.

The molecular weight of polymer is usually positively correlated with the physical properties of polymer solution (such as the viscosity of polymer solution, entanglement degree of molecular chain and rheological property) [57]. Its effect on fiber morphology is closely related to the properties above. Bilal Zaarour et al. used three kinds of PVDF with different molecular weights (Mw = 180,000, 275,000 and 530,000) to prepare electrospun fiber membranes. The fibers designed by PVDF with high molecular weight had thick diameters. When the molecular weight is high, the viscosity of the polymer solution is also high; the increase in the tension resistance of the jet from the spinneret results in the formation of large diameter PVDF fibers [58]. However, when the molecular weight is below the critical number, spray particles or bead-like fibers are easily formed because of Rayleigh instability [59].

When other parameters are constant, the concentration of polymer spinning solution can substantially affect the surface morphology of the fiber. Determining the suitable concentration of polymer spinning solution to prepare the best morphology fibers is also called the exploration of the spinning window. Xu et al. [60] mentioned that the polymer has a narrow spinning window in a particular solvent, and the spinning window of gliadin in HFIP is 15–20% (*w/v*). When the working fluid concentration is from 5% (*w/v*) to 20% (*w/v*), the morphology of the prepared products changes from particles to beads and then to a straight line. When the concentration continues to increase to 25% (*w/v*), the viscosity of the gliadin solution becomes excessively high. The spinneret becomes blocked by the surrounding semi-solid substances, thus preventing normal electrospinning.

The electrical conductivity of the solution is another important factor affecting fiber morphology. Proper electrical conductivity supplies the jet surface with an appropriate charge during electrospinning; thus, fibers with good morphology are formed. When the electrical conductivity increases in a specific range, the surface charge density of the jet and the Coulomb repulsion force between the jet surface and the layer also increases. In this situation, the enhancement of the bending whipping effect during electrospinning is conducive to the formation of fibers with a small diameter. However, the bending whipping of the jet is unstable under a strong electric field and forms fibers with uneven diameter distribution. Excessively high electrical conductivity hinders the formation of Taylor cone, and prevents spinning to normally proceed. Adulteration of salt compounds in the working fluid can increase the electrical conductivity to prepare fibers with good morphology. After adding NaCl to PCL spinning solution, Vince Beachley et al. [61] found that the diameter of fibers decreased with the increase in NaCl concentration. Under constant parameters, bead-like fibers could be observed when no NaCl was added in the working fluids. The diameter of the fiber can also be increased by increasing the electrical conductivity of the polymer solution through salt addition. Fuat Topuz et al. [62] used the self-polymerizing microporous polymer (PIM) solution to prepare fibers and found that the working fluid could be electrospun at a low concentration to form fibers after ammonium salt addition. However, the diameter of the fiber slightly increased possibly because the addition of ammonium salt had increased the viscosity of the polymer solution.

The dielectric constant of the solvent used in the polymer working fluid has an essential influence on the electrospinning process. Luo et al. found that electrospun PCL fibers with diameters less than 100 nm can be obtained when the dielectric constant of the solvent is 19 or higher. At low values, the products are fibers or residues with diameters in the range of submicrons to millimeters [63]. The applied voltage required for stable electrospinning increases with the increase in the dielectric constant of the solvent system. The jet becomes unstable when the dielectric constant of the selected solvent is extremely high.

Surface tension refers to the force applied on the surface plane per unit length [64] and has a particular influence on the electrospinning process and fiber morphology. In electrospinning, the Taylor cone can be formed only when the electrostatic repulsion on the surface of the droplet is greater than the surface tension, thus initiating the spinning process. High surface tension often requires a high voltage to break for a stable spin, thus hindering the formation of Taylor cone and affecting the normal spinning process [65]. The choice of solvent can directly determine the surface tension of the polymer solution. Yang et al. [66] systematically studied the effect of surface tension on the morphology of electrospun products by using PVP as a polymer model and ethanol, DMF, and MC as solvents. The results showed that different solvents lead to varying surface tensions. At fixed concentration, the bead-like fibers can be transformed into smooth linear fibers by using the solvent with low surface tension. When other conditions are fixed, the surface tension determines the upper and lower boundaries of the spinning window [67].

Viscosity is closely related to and determined by molecular weight and entanglement degree. High polymer concentration and large molecular weight indicate that the polymer chain has a high entanglement degree; in addition, the mobility of the chain in the solution is low, thus preventing the reordering of the chain and increases the viscosity of the solution [68]. Therefore, the effect of viscosity on fiber morphology is the same as that of concentration and molecular weight. Andrea Dodero et al. increased the viscosity by increasing the concentration of the working fluid and found that the uniformity and global structure of prepared electrospun chitosan fibers can be improved by increasing the solution viscosity [69]. The choice of solvent also affects the viscosity of the working fluid. Juliana et al. [70] used PI as a polymer template and selected four best solvents for PI (NMP, DMF, DMAc and DMSO) for electrospinning experiments. Large diameter PI fiber could be obtained by using the solvent with high viscosity. When the other parameters remain unchanged, the use of high-viscosity solvent extends the fiber beads. The linear distance between the beads and beads increases, and the fiber morphology changes from bead-like to spindle-like.

Solvent type determines many properties of the working fluid, such as dielectric constant, surface tension and viscosity. Solvent selection is important for the preparation of nanofibers with good morphology. The solubility of the polymer in the solvent and the boiling point of the solvent are two crucial considerations in the selection of solvents [71]. First, the polymer needs to have good solubility in the solvent; that is, the solvent molecule needs to overcome the intermolecular force in the polymer. In the electrospun polymer solution, mixed solvents can be used to control the fiber surface morphology to prepare smooth or porous fibers. Hoik Lee et al. [72] used mixed solvents to dissolve CA and studied the effects of different solvents on the surface morphology of CA fibers. DMF is a good solvent for CA, whereas acetone and MC are not suitable for CA. When other parameters are kept constant, the fiber with a smooth surface can be obtained efficiently by increasing the proportion of unsuitable solvents. Given that CA can only dissolve well in a good solvent, increasing the ratio of a not-good solvent reduces the space of CA diffusion in the solvent. Therefore, the viscosity of CA solution increases, and smooth fibers without beads can be prepared. When acetone and MC were selected as the mixed solvent of CA solution, the fiber surface was porous because the polymer could not exist uniformly in the solvent and could not spread correctly during spinning, thus resulting in rough and porous morphology. Second, the volatility of the selected solvent must also be considered for the preparation of fibers with good morphology. Highly volatile solvents easily cause spinneret blockage and thus should be avoided. Medium volatile solvents are beneficial to the spinning process and form bead-free fibers. Low volatile solvents are not conducive to spinning and hinder the solvent volatilization of the jet in the process of straight jet and bending whipping, thus easily producing bead-like fibers.

#### 2.3.2. Process Parameters

Process and system parameters can all affect the morphology of electrospun fibers. Process parameters are also known as operating parameters. Herein, the effects of voltage, flow rate, receiving distance and spinneret diameter on fiber morphology are summarized.

In electrospinning, the applied voltage directly determines the charge density of the jet surface and the Coulomb repulsion force. Increasing the voltage also increases the charge density on the jet surface, which in turn increases the Coulomb repulsion between the surface and the layer of the curved whipping section during spinning. Hence, the stretching effect of the jet is enhanced, and fibers with small diameters are formed. Seyed Jalaledin Najafi et al. [73] explored the effect of process parameters on the morphology of glass fibers (ethyl orthosilicate and PVP are the main polymer components). When the voltage was increased from 20 to 26 kV, the average diameter of glass fibers decreased gradually. When the voltage continued to grow to 28 kV, the average diameter of glass fibers increased. The increase in the average diameter of fibers with the applied voltage is also reflected in the study of Fuat Topuz et al. [74]. Their explanation for this phenomenon is that the increase in voltage also increases the flow of liquid at the spinneret, thus expanding the diameter of the fiber. This idea emphasizes the interaction between voltage and liquid flow. Another angle can be explored for this phenomenon. The increase in voltage accelerates the volatilization of the solvent during spinning. Hence, the jet does not have sufficient time to stretch, and the fibers with a large diameter are obtained. Therefore, the effect of voltage on fibers’ morphology must be considered within a specific critical value, and the influence of other parameters should not be neglected.

When other parameters remain unchanged, increasing the liquid flow rate during electrospinning will increase the diameter of the fibers but will produce fibers with poor surface morphology (fibers with spindles-like or bead-like) [75,76,77]. The increase in flow rate implies that many products are obtained in a fixed time, but the drying time of the jet in the corresponding spinning process is substantially shortened [71].

The receiving distance in the electrospinning process usually refers to the linear distance between the tip of the spinneret and the collector device. The effect of receiving distance on the fibers’ morphology is generally related to other parameters (voltage and solvent volatilization rate). After the receiving distance is increased within the critical range, the jet can be fully stretched in the bending and whipping process, and fibers with a small diameter can be obtained. When the increasing degree of the receiving distance exceeds the critical receiving distance, the electrostatic field force decreases, which is not conducive to the stretching of the jet. On the contrary, the fibers with a large diameter are obtained [78].

The diameter of the spinneret affects the diameter of the electrospun fiber, but the specific mechanism remains unclear. Until He et al. [79] used spinnerets with different diameters to prepare PEO nanofibers for rheological experiments and the shear flow model to analyze the effect of spinneret diameter on PEO fibers’ diameter. The liquid viscosity on the tip of the spinneret with a larger diameter is higher; hence, the essence of spinneret diameter on fibers’ morphology is the effect of fluid viscosity on fibers.

#### 2.3.3. Environmental Parameters

Environmental parameters are the secondary parameters that affect the morphology of electrospun fibers. The influence of environmental parameters on the morphology of electrospun fibers is closely related to other parameters. Huang et al. [80] studied the effect of temperature on the morphology of PVDF fibers and found that the diameter of PVDF fibers decreased and the fiber surface became rough with the increase in temperature. When the ambient temperature is increased appropriately, the surface tension and viscosity of the solution decrease; hence, the fiber with a small diameter will be formed. When a mixed solvent of DMF and acetone is used to dissolve PVDF, the high boiling point of DMF hinders the volatilization of low boiling point acetone, thus giving the fiber surface a ravine shape, which is evident at high temperatures. Cui et al. [81] also found that the fibers’ diameter decreased with the increase in temperature. Environmental humidity affects the fiber morphology mainly by influencing the solvent volatilization rate in the spinning process [82]. When the humidity is increased, the solvent volatilization on the jet surface is not complete and bead-like fibers with poor morphology are easily formed. Therefore, ambient temperature and humidity have a particular influence on fibers’ morphology.

### 2.4. Process Type of Electrospinning Technology

**Figure 5:**
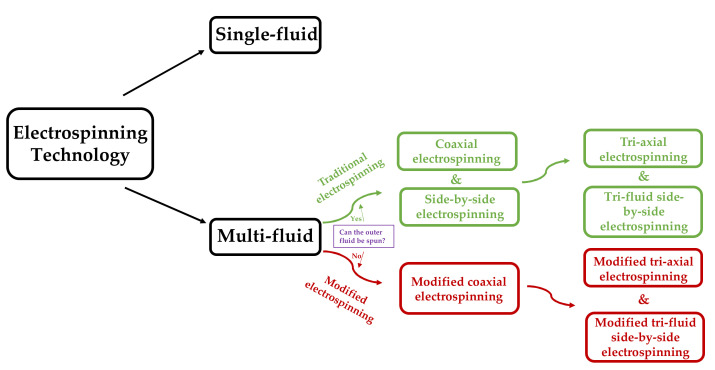
Classification of electrospinning technology.

With the in-depth understanding of electrospinning technology, electrospinning technology is continuously being upgraded and optimized. Figure 5 shows that according to the number of working fluids in the spinning process, the electrospinning process can be divided into single- and multifluid electrospinning technology. Multifluid electrospinning technology can further be classified according to the spinnable of the outer fluid into traditional and modified electrospinning technology. Traditional electrospinning technology can also be divided into coaxial and side-by-side electrospinning technology according to the spatial position of the working fluid. Different nanostructures can be prepared by different electrospinning processes, such as core–sheath [83] and Janus [84].

**Figure 6:**
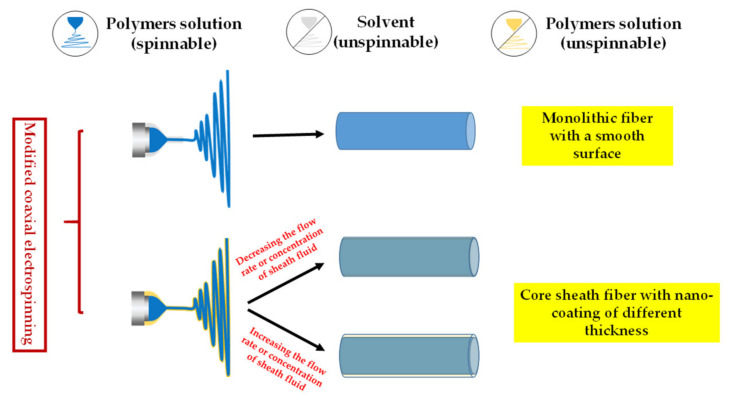
Modified coaxial electrospinning process.

In 2002, Loscertales, I. G. et al. [85] performed coaxial electrospraying experiments (sister technology of electrospinning) and prepared the core–shell nanoparticles for the first time. In the following period, coaxial electrospinning technology has received remarkable attention and development. From the traditional perspective of coaxial electrospinning, researchers believe that the interface between the core and sheath solution produces shear force due to contact friction and viscous dragging. When the shear stress caused by the viscosity of the sheath solution overcomes the interfacial surface tension between the core–sheath solution, the core–sheath fiber can be prepared stably [86]. Therefore, the viscosity of the sheath fluid should be higher than that of the core liquid, and the flow rate of the sheath fluid should be increased to ensure that the sheath liquid could completely cover the core liquid. Hence, the sheath solution must be spinnable prior to coaxial spinning to obtain the fibers with core–sheath structure. However, Yu et al. [87] broke this traditional cognition and used the non-spinnable solvent as the core-wrapping solution for the first time and successfully conducted the modified coaxial electrospinning experiment. After a series of experiments, the core–sheath fibers with a nano-coating layer structure were obtained when the non-spinnable polymer solution was used as the outer solution [88,89]. Figure 6 shows that when using pure solvent as a sheath solution for coaxial electrospinning, the spinning process is stable and a smooth surface monolithic fiber can be obtained. The external solvent can prevent the blockage of the spinneret, resist the negative influence of the external environment [90], and ensure a stable and continuous electrospinning process. In addition, the outer solvent needs to be a good solvent for the polymer being spun. A good solvent as outer fluid can adequately slow down the volatilization rate of the solvent in the working fluid and extend the stretching period of the jet under the action of the electric field to easily obtain high-quality fibers with small diameter and smooth surface. When the non-spinnable polymer solution is used as the sheath solution, the nanocoating with different thickness can be achieved by controlling the flow rate and concentration of the sheath. This discovery dramatically broadens the available polymer materials for electrospinning. At present, about 100 kinds of spinnable polymer materials have been discovered. However, many types of liquids are still not spinnable. With this technology, various liquids (solution, emulsion and suspension) can be used in electrospinning, thus fully utilizing the advantages of fiber membrane in various fields [91].

**Figure 7:**
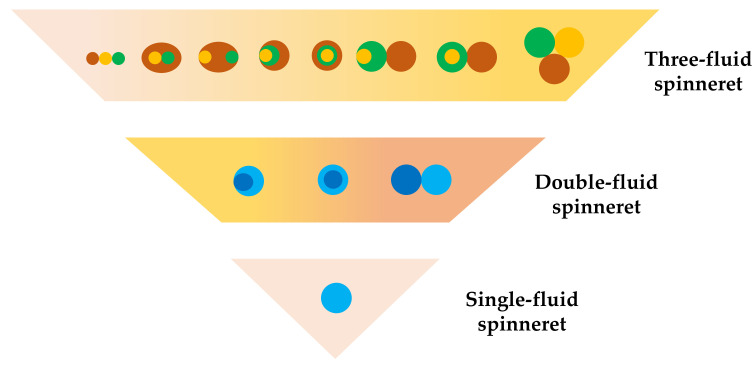
Types of spinnerets in electrospinning.

In the foreseeable future, electrospinning could be applicable in a broad range of working fluids. The key to updating this process lies in the design of the spinneret structure. Figure 7 simply summarizes the top view structure of the spinnerets. Fibers with complex nanostructures can be prepared through multifluid electrospinning and can be used in different fields, such as drug release [92,93,94,95,96,97,98,99], tissue engineering [100], lithium battery energy storage [101,102], antibacterial [103,104] and environmental remediation [105,106]. However, only few studies focused on the use of multifluid electrospinning to prepare functional fiber membranes for water treatment applications. Multifluid electrospinning shows a high research space and research value because it is suitable in preparing multistage nanofiber functional membranes with high mechanical properties and can carry various functional substances. This technology will occupy an important position in the field of water treatment in the future.

## 3. Electrospun Functional Fiber Membrane for Antibiotic Removal

The following conditions must be satisfied when using electrospun fiber membrane for water treatment. (1) The fiber membrane must have specific functionality. The preparation of electrospun functional membrane can be realized in three ways. Polymers with functions, such as adsorption, can be directly used to prepare electrospun functional nanofibers; the functional substances can be added into the working fluid to prepare the electrospun functional fiber membrane; the prepared electrospun fiber membrane can be endowed with membrane function by post-treatment techniques such as coating [107,108], heat treatment [109,110] and cross-linking [111,112,113,114]. (2) The prepared fiber membrane needs to be hydrophilic but insoluble in water. (3) The fiber membrane needs to have good mechanical properties and chemical stability. (4) The polymer for preparing fiber should be an environment-friendly material with certain biodegradability and does not cause secondary pollution to the environment.

### 3.1. Preparation Technology of Electrospun Functional Fiber Membrane

The preparation and treatment technology of electrospun functional membrane is mainly divided into pretreatment technology and post-treatment technology, or the combination of the two technologies.

**Figure 8:**
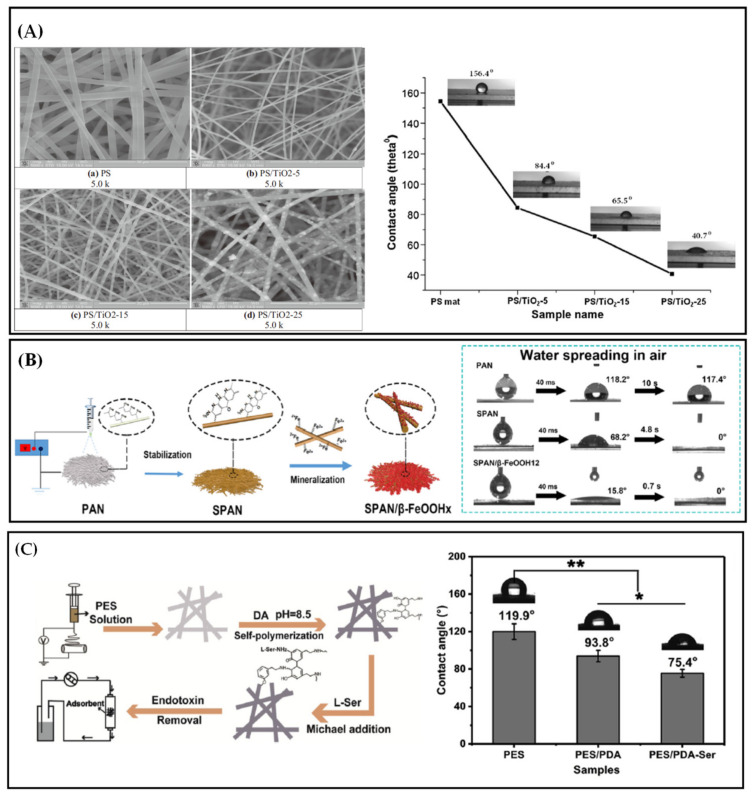
(**A**) SEM images and water contact angles of PS, PS/TiO_2_-5, PS/TiO_2_-15 and PS/TiO_2_-25 fiber membranes [115]; (**B**) preparation process and water contact angles of SPAN/β-FeOOHx nanofibers [118] and (**C**) preparation process and water contact angles of PES/L-Ser functional membranes [119].

Pretreatment mainly refers to the addition of functional substances to configure the electrospinning working fluid and provide it with certain functionality. The functional fiber membrane can be subsequently obtained by electrospinning technology. As shown in Figure 8A, TiO_2_ nanoparticles are directly added into the polymer solution of PS, and PS functional fibers containing TiO_2_ nanoparticles are prepared by single-fluid electrospinning technology for water treatment. When 25% TiO_2_ is added, the existence of TiO_2_ particles can be observed in the SEM images of PS/TiO_2_-25 fiber. The functional PS fiber membrane can be transformed into hydrophilic substance compared with the hydrophobic PS fiber without TiO_2_ nanoparticles. The hydrophilicity increases with the TiO_2_ content [115]. The preparation of functional membrane by directly adding functional substances into a spinning solution is a straightforward and efficient preparation technology. Whether the solvent used in the electrospinning working fluid destroys the structure of the functional particles or affects the usage of the functional particles should be fully considered when adding functional substances such as solid particles. For example, when acid solvents such as acetic acid are used, the solid particles with functional groups such as hydroxyl groups on the surface are not suitable to be mixed into electrospinning working fluid by pretreatment technology. In addition, the dispersion of functional solid particles in the working fluid should also be considered in the use of pretreatment technology. Low dispersion will clog the spinneret and affect the normal spinning process. The distribution of the functional particles mixed into the working fluid in the electrospun fiber will be uneven, and most of the functional sites will be wrapped in the fiber and fail to function because of the stretching of the jet and the volatilization of the solvent during electrospinning.

Solvents and functional substances with good compatibility must be selected when using pretreatment technology to prepare electrospun functional fiber membrane. The instability of the electrospinning process caused by functional substances precipitation can be avoided by selecting appropriate mixing concentration. In addition, the dispersion uniformity of functional substances in the final fiber product can be solved by using multifluid electrospinning technology. The functional substances can be mixed into the sheath, and the fiber with appropriate sheath thickness can be obtained by controlling the concentration and flow rate of the sheath [88,89] to ensure that the functional substances can fix in the sheath instead of being fully wrapped, and a large number of functional sites are exposed on the surface of the functional membrane for full utilization.

Post-processing indicates that the prepared electrospun fiber membrane is endowed with function through a series of membrane post-processing technology. Post-treatment technologies include physical (ultrasonic cracking, physical vapor deposition or plasma treatment) and chemical modifications (oxidation, hydrolysis, grafting and ammonolysis) [116]. Although physical modification is widely used, it cannot completely change the properties of the fibers in the deep layer of the membrane. By contrast, chemical modification can improve the properties of the internal fibers in the membrane because of its wide adaptability and incomparable advantages over physical modification. With post-treatment technology, the functional substances can be loaded on the fiber surface or directly endowed with the fiber functional groups. Before being loaded with functional substances, the membrane must be pretreated, such as calcination treatment or surface coating with a sticky coating such as PDA [117]. In Figure 8B, the electrospun PAN nanofiber membrane was used as the substrate for water treatment. After calcination, the PAN molecular chain was cyclized, dehydrogenated and oxidized, which made it easier to combine with Fe^3+^. The functional fiber membrane SPAN/β-FeOOHx with β-FeOOH nanoparticles uniformly loaded on the membrane surface was successfully prepared by heat treatment immersion method. The hydrophobic PAN membrane was transformed into a hydrophilic membrane (sPAN) after calcination, and the hydrophilic effect was significantly improved by loading β-FeOOH [118]. The use of PDA as a viscous coating has also achieved good results. As shown in Figure 8C, PES fiber membrane was prepared by electrospinning, and the serine (L-Ser) was fixed on the surface of fibers using PDA as a viscous coating to prepare the PES/L-Ser functional membrane. The PDA viscous coating can remarkably improve the hydrophilicity of the fiber, and the fiber is completely transformed into a hydrophilic membrane after functionalization [119]. The treated functional membrane must be hydrophilic to facilitate contact with antibiotics and other pollutants in water. Hence, the advantage of hydrophobic coatings such as epoxy resin [120,121] in water treatment is weakened considerably.

The functional substances on the functional membrane prepared by post-treatment technology are entirely exposed on the surface of fibers and coated evenly. With the advantage of a large specific surface area, the role of fibers in removing water pollutants can be realized. The full coverage of hydrophilic functional substances on the surface of fibers broadens the channels for water treatment with some hydrophobic polymers with good mechanical strength. Compared with the functional substances loaded on the surface, the risk of shedding can be greatly reduced by using the post-treatment technology to endow the functional groups of the fibers directly. Sol An et al. [122] demonstrated an effective chemical method for the functionalization of cellulose nanofibers using sulfhydryl groups. First, the electrospun cellulose acetate nanofiber membrane was deacetylated with 0.05 m NaOH aqueous solution to obtain cellulose nanofibers, then the membrane was added into the DMAc mixed solution of DTDPA and CDI, then vigorously stirred at 80 °C for 22 h, and the esterification of hydroxyl with DTDPA was carried out. The sample was further treated with AmTG aqueous solution at room temperature for 3 h to ensure that the disulfide bond was also reduced and broken. Sulfhydryl functionalized cellulose nanofibers were finally obtained.

**Figure 9:**
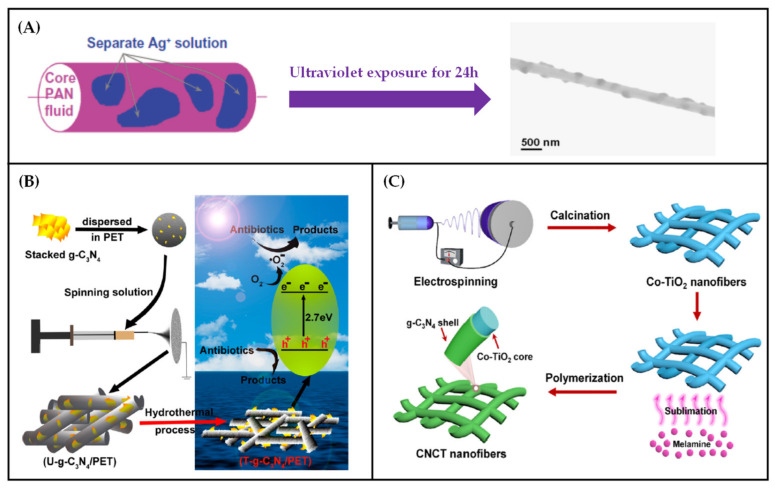
(**A**) The process of converting Ag^+^ into Ag nanoparticles on the surface of PAN fiber by UV radiation [123]; (**B**) the preparation process of T-g-C_3_N_4_/PET nanofibers and the mechanism of degradation of antibiotics under solar irradiation [124] and (**C**) flow chart of preparation of soft CNCT nanofiber films [125].

The preparation technology of electrospun functional fiber membrane can also combine pretreatment technology with post-treatment technology. Table 3 summarizes the specific preparation parameters of electrospun functional membranes prepared by the comprehensive use of pre- and post-treatments. In this method, the precursor substance is mixed into the spinning working fluid and then transformed into functional substance loaded on the surface of fiber through a series of treatments. In Figure 9A, Yu et al. [123] used 10% (*w/v*) AgNO_3_ solution as sheath solution and 15% (*w/v*) PAN DMAc solution as core solution, successfully prepared PAN nanofibers with Ag^+^ on the surface by modified coaxial electrospinning technology, and then they irradiated the fibers under a UV lamp with a wavelength of 254 nm for 24 h to ensure that the Ag^+^ on the fiber surface was reduced to Ag nanoparticles and attached to the surface of fibers. Compared with the pretreatment technology of directly adding Ag nanoparticles to the spinning working fluid, this functional membrane preparation technology dramatically reduces the functional waste caused by the embedding of silver nanoparticles in fibers. A method to reduce the effect of this phenomenon is also shown in Figure 9B. First, the g-C_3_N_4_ functional particles were dispersed in the HFIP solution of PET, and the functional fiber membrane was then prepared by single-fluid electrospinning. After the functional fiber membrane is heat-treated with an alkaline aqueous solution at 65 °C for 1.5 h, the embedded g-C_3_N_4_ reappears on the surface of fibers, which is called T-g-C_3_N_4_/PET membrane. Compared with the untreated film, the photodegradation efficiency of SQX was significantly improved [124].

Through the simultaneous use of pretreatment and post-treatment technology, the fiber membrane can also be given different functions, which can be used for water treatment. In Figure 9C, the researchers first mixed the gel solution of TiO_2_ into the spinning working fluid of PVP using the pretreatment technique and a monolithic mixed functional fiber membrane Co-TiO_2_ was prepared by using single-fluid electrospinning technology. At this time, the membrane had the related function of TiO_2_. The surface of fibers was then coated with g-C_3_N_4_ nanosheath by in-situ thermal polymerization using melamine as the precursor. A core–shell CNCT functional nanofiber membrane with dual functions was prepared, which can degrade both TC and *Escherichia coli* at the same time [125].

The above technologies can give the membrane new function, improve the hydrophobicity, mechanical properties and chemical stability of the membrane, and make the fiber membrane suitable for the treatment of water pollutants [126].

### 3.2. Application of Electrospun Functional Fiber Membrane for Antibiotic Removal

#### 3.2.1. Adsorption

**Figure 10:**
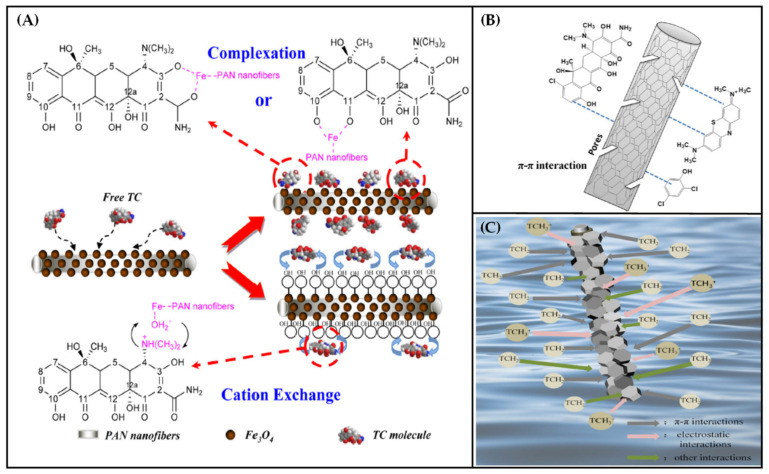
(**A**) Adsorption mechanism of TC on Fe_3_O_4_/PAN composite nanofiber membrane [130]; (**B**) adsorption mechanism diagram of TC, 2,4-DCP, MB on PI-based carbon nanofiber membrane [131] and (**C**) adsorption mechanism diagram of TC on ZIF-8/PDA/PAN nanofiber membrane [132].

Adsorption mainly refers that the existence of one or more dissolved pollutants (antibiotics in this case) in the fluid phase and the addition of the adsorption material causes the dissolved contaminants to be transferred from the liquid phase to the solid surface [129]. The main steps include: (1) solute transports in the fluid phase around the adsorption material; (2) membrane diffusion; (3) pore diffusion and (4) interaction between the adsorbed material and the porous structure. Absorption can be divided into physical and chemical types according to the principle of action. Physical adsorption is mainly manifested by the pore adsorption of porous materials and second-order interactions between pollutants and functional groups on the surface of adsorbents (hydrogen bond interaction, electrostatic interaction and hydrophobic interaction). Chemical adsorption is mainly the exchange or transfer of electrons between pollutants and adsorbents to produce chemical bonds with strong binding force. As shown in Figure 10A, the adsorption of antibiotic molecules by electrospun functional fiber membrane can be realized by complexation (the relationship between the metal bonds of functional substances on the membrane surface and the corresponding functional groups (such as carbonyl groups) of antibiotic molecules) and cation exchange (the interaction of functional groups between the membrane surface and antibiotic molecules with the same positive charge) [130]. In Figure 10B, adsorption can also be achieved by the π bond interaction between the surface of the functional fiber and the antibiotic molecules [131]. Adsorption can be achieved through the electrostatic attraction between the surface of the functional fiber and the antibiotic molecules with different charge functional groups (Figure 10C) [132]. Complexation and cation exchange belong to chemical adsorption, which is irreversible and forms a strong interaction, but it is limited to monolayer cover; π bond interaction and electrostatic attraction belong to physical adsorption, which is reversible and mainly involves the interaction between van der Waals force and the second order, and the interaction force is weak.

As a widely used technology for water treatment, adsorption has the advantages of low energy consumption, low cost, high efficiency, few byproducts, simple operation and easy scale [133]. In the treatment of wastewater containing trace pollutants such as antibiotics (ng/L-mg/L), adsorption technology has extremely high competitiveness and efficiency. The common adsorption materials that can be used for adsorption include activated carbon [134,135], boron nitride [136], lignocellulose [137], porous materials [138], graphene [139], montmorillonite [140], cerium oxide [141] and iron oxide [142,143]. Most of the adsorption materials are in the form of powder and not conducive for recovery during wastewater treatment. In addition, this method can easily cause secondary pollution to the environment. The electrospun fiber membrane as a carrier loaded with powder adsorption materials can better solve this problem. The surface of the electrospun fiber membrane can carry a large number of functional groups and provide more adsorption sites. Its larger surface area provides an excellent substrate platform for the loading of functional adsorption materials, which has a high advantage for the adsorption of antibiotic molecules. Table 4 shows that electrospun functional fiber membranes have achieved good results in adsorbing antibiotics in water, and most systems have relatively high antibiotics adsorption capacity. However, the operating environment to achieve the best adsorption effect is mainly an acidic environment, which is a serious obstacle to practical application. It is worth noting that most electrospun functional fiber membranes adsorbed with antibiotics used NaOH solution as a desorption agent to realize the reusability test. The main reason is that the adsorption effect of these electrospun functional fiber membranes is very poor at high pH, and the use of alkaline solution can achieve a better desorption effect.

Montmorillonite is a common natural clay mineral material with a layered structure and large cation exchange capacity [144,145] and has attracted attention as an antibiotic adsorbent [146,147,148]. Electrospun CA nanofiber membrane impregnated with montmorillonite was developed to remove CIP antibiotics from wastewater [149]. CIP adsorption by the functional fiber membrane is affected by the pH value of the water environment because CIP has different ionization forms at different pH values: when pH < 6.1, CIP exists mainly in the cationic form; when 6.1 < pH < 8.89, CIP exists in the amphoteric form; and when pH > 8.89, CIP exists in the anionic form. At different pH values, the functional groups on the surface of the functional membrane will also be protonated or deprotonated to carry the charge. By measuring the zero-charge potential pH_PZC_ = 7 of the functional membrane (at a specific pH value, the surface of the functional material immersed in water has zero net charge, this pH value is called zero charge point [150]), the electrostatic interaction between functional fiber membrane and antibiotic molecules can be analyzed concretely. However, this study finally concluded that 6 is the best adsorption pH point. Since cation exchange is the usual adsorption mechanism of this kind of natural clay minerals [151], both antibiotics and adsorbent have cations under these conditions, and cation exchange occurs between montmorillonite layers, which significantly increases the adsorption rate and adsorption capacity.

Metal–organic framework (MOF) materials are highly ordered porous crystal hybrid materials composed of metal clusters and organic connections of multifunctional groups [152,153,154]. As an excellent adsorbent, MOFs has also attracted wide attention in the field of antibiotic adsorption [155,156,157,158,159]. Aowen Li et al. [13] demonstrated that electrospun PVA/SiO_2_ fiber membrane modified with dopamine could be loaded with MOFs materials for adsorption of antibiotics in water. The adsorption mechanism of MOFs to antibiotics mainly belongs to physical adsorption and has extremely high adsorption capacity without changing the pH value of the water environment. With the help of the advantages of high specific surface area, high porosity and good chemical toughness of electrospun fiber membrane, it shows excellent adsorption effect of antibiotics.

The use of electrospun functional fiber membrane as an antibiotic adsorbent in water bodies has excellent advantages. However, the overall research is still in its infancy, has not been greatly expanded, and requires a long course to reach practical application. As a technology to transfer the target pollutants in the liquid phase to the solid phase, adsorption will not produce other harmful substances. However, the target pollutants have not been eliminated, and the contaminants might fall off from the surface of the adsorbent. A solution is to develop the use of adsorbent materials with robust adsorption mechanism such as chemical adsorption as the primary adsorption mechanism. The use of iron oxides as chemical adsorbents for antibiotics has achieved remarkable results [160,161]. Amorphous hydrated iron oxide and γ-Fe_2_O_3_ can adsorb LEV and TC molecules by bridging double chelation and complexation, respectively [162]. Two typical amorphous and crystalline iron oxide minerals, iron hydrate and goethite, can chemisorb TC molecules to form stable inner spherical complex [163]. This kind of metal oxides has a bright future in the field of antibiotic removal in water bodies in the future.

#### 3.2.2. Photocatalysis

Photocatalytic technology belongs to advanced oxidation technology. The degradation forms of photocatalysis include direct photolysis, photo/chemical reagent degradation (ozone and H_2_O_2_), photo-Fenton degradation (Fe^2+^/H_2_O_2_/light) and photoresponsive semiconductor degradation [170]. Photochemical reagents degradation (ozone and H_2_O_2_) belongs to indirect photolysis. Chemical reagents absorb light and decompose to produce highly active substances, such as singlet oxygen (^1^O_2_), hydroxyl radical (·OH) and alkyl peroxyl radical (OOR) [171]. Photo-Fenton degradation and photoresponsive semiconductor degradation belong to self-sensitized photolysis. The catalysts absorb light energy to the excited state, transfer the energy to the ground state ^3^O_2_, H_2_O and produce reactive oxygen species, such as ^1^O_2_ and ·OH, which mediate the degradation of pollutants [171]. Photo-Fenton degradation requires the addition of H_2_O_2_ and relies on the Fe (II)/Fe (III) redox cycle to activate H_2_O_2_ to produce active substances, such as hydroxyl radical (·OH) and high valent iron, which degrade organic or inorganic compounds in the presence of ultraviolet (UV) rays [172]. The primary reaction is shown in the following formula [173]:

(3) $$Fe^{2+}+H_{2}O_{2}\to Fe^{3+}+OH+OH^{-}$$

(4) $$OH+H_{2}O_{2}\to \cdot OH_{2}+H_{2}O$$

(5) $$2\cdot OH\to H_{2}O_{2}$$

The main steps involved in photoresponsive semiconductor degradation are as follows [174]: (1) when the semiconductor is irradiated by light, the photons whose energy is greater than the semiconductor bandgap excite the electrons in the semiconductor valence band into the conduction band; (2) the semiconductor valence band creates holes, which can oxidize the donor molecules and can further react with water to produce·OH, and act as a strong oxidant to degrade pollutants and (3) the electrons in the conduction band can also react with dissolved oxygen species to form superoxide ions, which promotes the redox reaction. The use of photocatalytic technology to treat wastewater has the advantages of high efficiency, environmental protection and no secondary pollutants [175], thus providing increased benefits in the degradation of antibiotics and other water pollutants.

**Figure 11:**
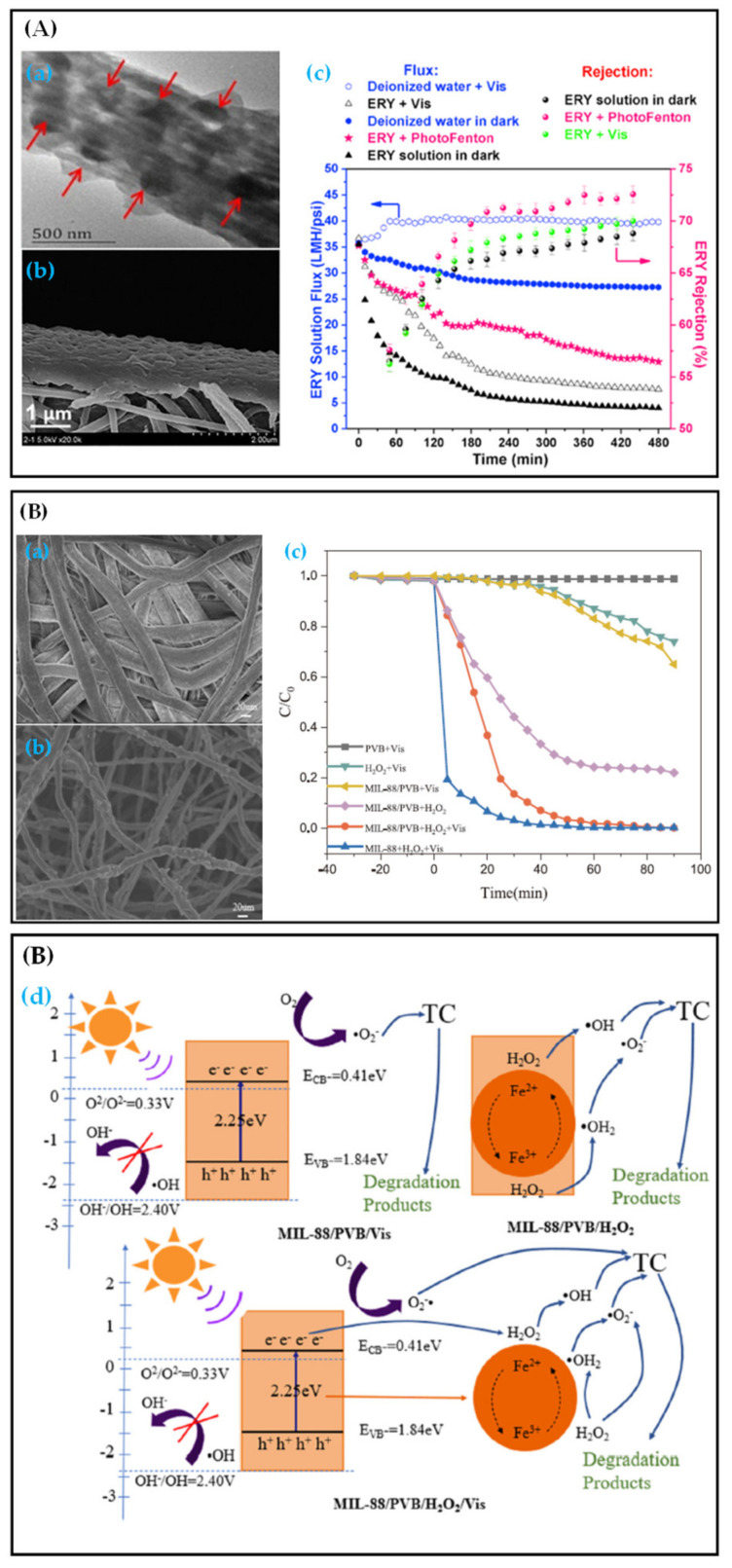
(**A**) CS/PAN@FeOOH/g-C_3_N_4_ photo-Fenton membrane [181]: (**a**) SEM image of single PAN@FeOOH/g-C_3_N_4_ nano-support fiber, (**b**) SEM image of cross-sectional structure and (**c**) water flux and rejection of ERY by the CS/PAN@FeOOH/g-C_3_N_4_ membrane; (**B**) MIL-88/PVB photo-Fenton membrane [183]: (**a**) SEM image of PVB fibers, (**b**) SEM image of MIL-88/PVB fibers, (**c**) degradation activity of TC in different conditions and (**d**) the mechanism schematic diagram of the reaction process.

Akaganeite (β-FeOOH) has a tetragonal structure composed of double chains with shared octahedrons [176]. Iron atoms firmly bind to the skeleton to form a tunnel, which belongs to semiconductor materials with a bandgap of 2.12 eV [177]. The synthesis condition of β-FeOOH is generally acidic (pH < 5). Hence, a certain amount of outer frame anions such as halogen ions are needed in its structure to balance the extra protonation of oxides in octahedral iron. Its tunnel structure also popularizes β-FeOOH in the field of catalysis [178]. When β-FeOOH is used as the photocatalyst, substances such as H_2_O_2_ should be used as external substances to produce the photo-Fenton reaction. β-FeOOH or related β-FeOOH composite particles have been used to catalyze the degradation of drugs such as antibiotics. Li et al. [179] compounded β-FeOOH with Fe_3_O_4_ to prepare magnetic nanocomposite particles. SMX was degraded by adding peroxymonosulfate, and the degradation rate of SMX in 30 min reached 91%. Luo et al. [180] used palygorskite loaded with β-FeOOH to photocatalytic remove MTZ by adding H_2_O_2_. The degradation rate of MTZ reached 92.8% within 180 min. Although β-FeOOH has achieved good results in the catalytic degradation of antibiotics and other drugs, the recovery and reuse of the catalyst remain a problem. The preparation of photo-Fenton fiber membrane can solve this problem well. Almost no study was conducted on the preparation of photo-Fenton fiber membrane using β-FeOOH. However, Zheng et al. [181] studied and synthesized a kind of FeOOH/g-C_3_N_4_ submicron particles (the type of FeOOH is not specified), which were put into the PAN fiber membrane by electrospinning pretreatment technology (Figure 11(Aa)). A layer of CS was then uniformly coated on the surface of fibers to prepare a highly hydrophilic CS/PAN@FeOOH/g-C_3_N_4_ membrane (Figure 11(Ab)). As shown in Figure 11(Ac) [181]: under dark conditions, the water flux of simple ERY decreased from 35.6 to 9.8 LMH·psi^−1^ in 120 min, and then reduced to 3.9 LMH·psi^−1^ in 120–480 min. The fouling of ERY and water pressure together make the effective porosity of the membrane decrease, resulting in the decline of water flux. Under the light condition, the water flux decline rate of ERY on CS/PAN@FeOOH/g-C_3_N_4_ membrane was lower than that in the dark state, and the water flux decreased to 7.6 LMH·psi^−1^ in 480 min. This finding shows that CS/PAN@FeOOH/g-C_3_N_4_ membrane has a specific effect on the degradation of ERY under light conditions and alleviates the blockage of membrane pores caused by ERY. When H_2_O_2_ was added, the water flux decreased slowly to 12.6 LMH·psi^−1^ in 480 min, indicating that the CS/PAN@FeOOH/g-C_3_N_4_ membrane can effectively degrade ERY, through the photo-Fenton reaction. This conclusion can also be obtained from the rejection rate of ERY under different conditions.

MOFs have good adsorption and can be used similarly to β-FeOOH as an efficient photocatalyst for antibiotic degradation by the photo-Fenton reaction. An iron-based MOF material (MIL-88/PVB) synthesized by the hydrothermal method could degrade TC by the photo-Fenton process after the addition of the sulfuric acid group [182]. This process was further updated, and the PVB electrospun fiber membrane was used as the substrate. As shown in Figure 11(Ba,Bb) [183], MIL-88 nanoparticles were successfully loaded on the surface of PVB fibers, which led to the transformation of PVB fibers’ surface from smooth to rough. MIL-88/PVB photo-Fenton fiber membrane can effectively degrade TC by the photo-Fenton reaction. However, the degradation rate of TC by photo-Fenton was slightly lower than that of pure MIL-88 particles, possibly because of the package of the PVB layer (Figure 11(Bc)). Under the condition of photo-Fenton reaction, MIL-88/PVB photo-Fenton membrane could produce more ·OH and O^2-^ through photosensitive reaction and Fe (II)/Fe (III) redox cycle (the primary reaction), and the efficiency of TC degradation was significantly improved (Figure 11(Bd)).

The photocatalytic fiber membrane can be prepared by loading the photosensitive semiconductor materials on the surface of fiber membrane. Compared with the photo-Fenton fiber membrane, the former can degrade the antibiotics without adding chemical reagents. The number of studies on the degradation of antibiotics by photocatalytic fiber membrane increases annually [184,185].

Titanium-based semiconductors received research attention as typical photocatalytic semiconductors. TiO_2_ has been widely used in photocatalytic degradation of organic pollutants in water but has some shortcomings, such as a wide bandgap (3.2 eV), low utilization of visible light, easy recombination of electron holes, the short lifetime of photogenerated carriers and low quantum efficiency [186]. The photocatalytic ability of semiconductor photocatalysts can be improved by broadening the spectral absorption capacity of semiconductor photocatalysts, increasing the separation and migration rate of photogenerated electrons, and preventing the recombination of photogenerated electrons and holes [187]. The preparation of TiO_2_ into composite materials containing heterostructures is a general method to improve photocatalytic activity. Heterojunctions with strong redox properties can generate built-in electric fields around the interface, effectively separate photogenerated electron pairs, and improve photocatalytic performance [188,189]. Electrospun nanofiber membrane provides a good platform for the preparation of heterojunction structure. Bao et al. [190] used electrospinning technology to prepare PAN nanofiber membrane and then combined calcination and solvothermal method to load TiO_2_ nanoparticles and BiOCl nanosheets on the surface of PAN carbon nanofibers (CNFs). The TiO_2_ nanoparticles form a strong redox heterojunction with BiOCl nanosheets on the surface of fibers. In 210 min, the photodegradation efficiency of TC, CIP, NFC and OTC could reach 82%, 94%, 90% and 92%, respectively.

**Figure 12:**
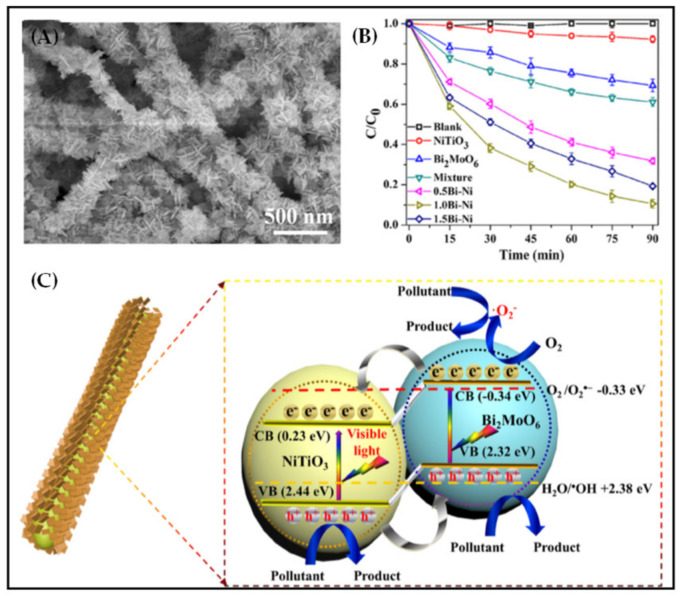
(**A**) SEM image of NiTiO_3_/Bi_2_MoO_6_ nanofiber membrane (1.0 Bi-Ni); (**B**) degradation efficiency of TC and (**C**) possible photocatalytic mechanism for TC degradation over NiTiO_3_/Bi_2_MoO_6_ nanofiber membrane (1.0 Bi-Ni) [191].

Compared with TiO_2_, titanium-based perovskite oxide (NiTiO_3_) had a suitable bandgap (2.18 eV) and unique sunlight lighting ability. However, its photocatalytic performance was greatly limited because of its low carrier separation ability. NiTiO_3_ nanofiber membrane was prepared by electrospinning and calcination and then uniformly loaded with Bi_2_MoO_6_ by the solvothermal method [191] (Figure 12A). As shown in Figure 12B, the TC degradation efficiency of NiTiO_3_/Bi_2_MoO_6_ nanofiber membrane (1.0 Bi-Ni) was 11.6 times higher than that of pure NiTiO_3_ nanofiber membrane. The antibiotic degradation activity of the physical mixture of NiTiO_3_ and Bi_2_MoO_6_ was significantly lower than that of NiTiO_3_/Bi_2_MoO_6_ heterojunction nanofiber membrane, indicating the good interfacial contact between NiTiO_3_ and Bi_2_MoO_6_ achieved by loading on the fiber surface. The main active substances for photocatalytic degradation of TC by NiTiO_3_/Bi_2_MoO_6_ were holes and superoxide radicals, which was similar to the TiO_2_/BiOCl. The close contact and matched band arrangement on the fibers could effectively promote the transfer and separation of carriers, and significantly improve the photocatalytic degradation of TC (Figure 12C).

#### 3.2.3. Biodegradation

Biotechnology is a promising method to degrade antibiotic pollutants in water. Enzyme biodegradation of antibiotic pollutants in water shows remarkable advantages [192,193], such as high specificity and selectivity of catalytic reaction [194], no toxic byproducts during degradation, environmental friendliness, simple operation and mild reaction environment [195]. The common enzymes used to degrade antibiotics are mainly oxidoreductase, including tyrosinase, peroxidase (lignin peroxidase, manganese peroxidase and multifunctional peroxidase) and laccase [196]. Among them, laccase is a kind of copper-containing polyphenol oxide that widely exists in red or white-rot fungi and can catalyze the degradation of various pollutants through single electron transfer in the presence of molecular oxygen [197,198]. However, free laccase is limited by the following factors, including difficult recovery, low repeatable utilization, poor adaptability and tolerance to the environment, high cost and limited reaction stability [199]. Exploring the immobilization of laccase is the key to solve this problem. Magnetic nanoparticles (Fe_3_O_4_) have been used as carriers and were modified to load laccase with good results. A functional coating with wealthy amino groups (chitosan [200,201], DAS and amino-functionalized ionic liquids [202]) or sulfur groups (GMA-S-SH [203]) is usually coated on the surface of Fe_3_O_4_. Laccase can be immobilized on the surface of Fe_3_O_4_ by the covalent bonding between laccase and the coating or immobilized on the surface of Fe_3_O_4_ particles by the metal affinity adsorption of laccase molecules with Cu^2+^ (metal ions are chelated on the surface of Fe_3_O_4_ particles by forming hydroxyl or carboxyl groups) [204]. Immobilization improves the stability of laccase, and the magnetic Fe_3_O_4_ particle carrier is also convenient for using the external magnetic field to recover the bioactive catalytic system and enhance the repeatable utilization of laccase. However, the bioactive catalytic system prepared with magnetic particles, as laccase carrier is still granular and has some defects such as agglomeration. Recovery in a large-scale application remains difficult. Hence, further exploration about the immobilized enzyme carrier is still necessary.

**Figure 13:**
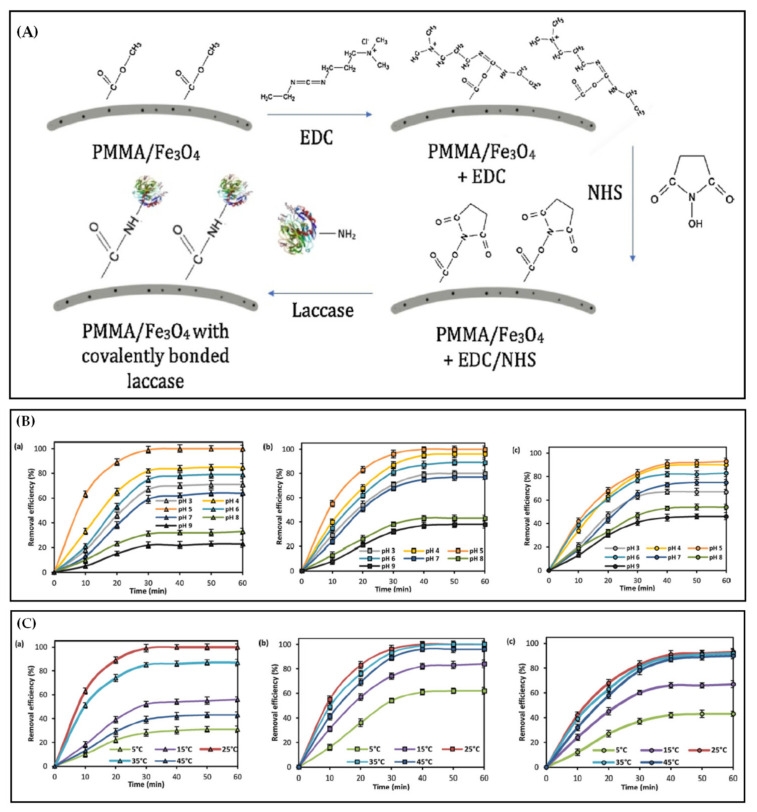
(**A**) Schematic of functionalization of PMMA/Fe_3_O_4_ nanofibers by the EDC/NHS method and covalent immobilization of laccase. (**B**) Effect of pH value on tetracycline removal by free enzyme (**a**), covalently bound laccase (**b**) and encapsulated laccase (**c**). (**C**) Effect of temperature on tetracycline removal by the free enzyme (**a**), covalent bound laccase (**b**) and encapsulated laccase (**c**) [196].

Electrospinning can be used to introduce enzymes in functional fibers by wrapping them inside or fixing them on the surface (adsorption, cross-linking or covalent bonding) for antibiotic degradation [205,206,207]. The enzyme-containing functional fiber membrane prepared by electrospinning can fully utilize the advantages of high specific surface area and large porosity of the membrane. The enzyme immobilized on the fiber surface has high stability and reusability and can be used to effectively degrade antibiotics in water. In Figure 13A, PMMA/Fe_3_O_4_ with covalently bound laccase functional fiber membrane was prepared by post-treatment technology (the PMMA/Fe_3_O_4_ fiber membrane was soaked in EDC and NHS to form a specific functional group and then combined with laccase molecules to form a covalent bond on the fiber surface) and used to degrade TC in water. PMMA/Fe_3_O_4_ with encapsulated laccase functional fiber membrane was also prepared by pretreatment (adding laccase to the working fluid) as a control group.

pH and temperature are two important factors that determine the activity of enzyme reaction. All forms of laccase have the highest catalytic degradation efficiency of TC under weak acidity at pH 5. Compared with free laccase (Figure 13(Ba)), PMMA/Fe_3_O_4_ with covalently bound laccase (Figure 13(Bb)) and PMMA/Fe_3_O_4_ with encapsulated laccase (Figure 13(Bc)) functional fiber membranes have a more comprehensive range of pH application. Especially the degradation rate of TC by PMMA/Fe_3_O_4_ with encapsulated laccase functional fiber membrane is still more than 40% under alkaline conditions. The carrier material PMMA and magnetic nanoparticles have a good protective effect on laccase, which can stabilize the structure of laccase and improve the tolerance of laccase in the harsh reaction environment. A study on the effect of temperature on the TC degradation of different forms of laccase revealed that 25 °C was the best reaction temperature for all forms of laccase. The laccase immobilized on the nanofiber membranes is more resistant to high temperature than the free laccase (Figure 13C). This phenomenon can be explained from two aspects. (1) The interaction between laccase and PMMA/Fe_3_O_4_ carrier can stabilize the structure of laccase and protect the enzyme from external conformational changes. (2) PMMA/Fe_3_O_4_ reduces the adverse effect of high temperature on enzyme activity and protects the biomolecules from heat inactivation [196].

Compared with the free enzyme, the fiber immobilized enzyme has greater advantages in storage, pH tolerance, thermal stability and reusability. However, the activity of an enzyme inevitably decreases when it is immobilized on the electrospun fiber membrane. Palanivel Sathishkumar et al. [198] immobilized laccase on PLGA fiber and found that the relative activity of immobilized laccase is only 82% of that of free laccase. Therefore, the technology of enzyme immobilization on fiber surface without damaging its activity remains a fundamental research direction that must be explored.

## 4. Conclusions and Prospect

Electrospinning is a general method for nanofiber preparation. After years of continuous improvement and development, this technology has shown great advantages as a simple, one-step method for polymer nanofibers. Different target fibers can be prepared by changing the system, process and environmental parameters of the electrospinning process, thus implying the high flexibility and practicability of this method in antibiotic degradation. With the continuous development and modification of this technology, the number of polymer materials that can be used for electrospinning has increased. However, the choice of polymers for antibiotic degradation in water is still limited. Hydrophilicity and mechanical properties are the key factors that limit the use of polymers. Hydrophilic polymer materials often have low mechanical properties, those with good mechanical properties are inferior hydrophilic, and the number of polymer materials with both characteristics is low. Further exploration and the development of modification technology are critical.

This paper reviewed the preparation technology of electrospun functional fiber membrane. Pre- and post-treatments ensured the perfect application effect of these materials in antibiotic degradation. The hydrophilicity and mechanical properties of the treated electrospun membrane were improved in varying degrees. Compared with those under post-treatment, the electrospun functional fiber membranes prepared by pretreatment have many defects because most of the functional materials are wrapped by the polymer layer, thus obscuring their effects. The acceptance of post-treatment technologies is highly extensive. Chemical post-treatment deeply changes the fiber properties compared with the physical one, and new functional degradation antibiotics are introduced on the fiber surface. The electrospun functional fiber membranes prepared by combine pretreatment and post-processing have more functional degree and a better application effect than those prepared using the single treatment.

The application of the electrospun functional fiber membrane in the adsorption, photocatalysis and biodegradation of antibiotics has achieved certain results. However, this field is still in its infancy. Problems such as membrane fouling, loading rate of functional substances, the photocatalytic activity of functional membrane and the decrease of enzyme activity after loading in biological treatment must be urgently solved. In the future, multifluid electrospinning can be used to develop multistage structural electrospun functional fiber membranes loaded with different functional substances. The use of multistage interaction of related substances to improve the performance of functional membranes could be an excellent solution.

## Figures and Tables

**Table 1:** Classification and some physicochemical properties of common antibiotics.

Classification	Stands for Antibiotics (CAS Number)	Molecular Formula	Solubility (mg/L)	Dissociation Constant pK ^a^	Predicted Critical Effect Concentration (ng/L) ^b^
Aminoglycosides	Gentamicin (1403-66-3)	C_21_H_43_N_5_O_7_	1 × 10^5^	9.0	1.8 × 10^8^
β-Lactam	Amoxicillin (26787-78-0)	C_16_H_19_N_3_O_5_S	3.43 × 10^3^ (25 °C, in water)	3.2, 11.7	1.9 × 10^14^
	Carbenicillin (4697-36-3)	C_17_H_18_N_2_O_6_S	451		
	Oxacillin (66-79-5)	C_19_H_19_N_3_O_5_S	13.9	2.72	
	Piperacillin (61477-96-1)	C_23_H_27_N_5_O_7_S	119		350,026
	Phenoxypenicillin (87-08-1)	C_16_H_18_N_2_O_5_S	240 (pH = 1.8, in water)	2.79	31,117
Chloramphenicol	Chloramphenicol (56-75-7)	C_11_H_12_Cl_2_N_2_O_5_	2500 (25 °C, in water)		8.1 × 10^6^
Fluoroquinolones	Ciprofloxacin (85721-33-1)	C_17_H_18_FN_3_O_3_	30,000 (20 °C, in water)	6.09	1.9 × 10^7^
	Danofloxacin (112398-08-0)	C_19_H_20_FN_3_O_3_			
	Enoxacin (74011-58-8)	C_15_H_17_FN_4_O_3_	3430		
	Enrofloxacin (93106-60-6)	C_19_H_22_FN_3_O_3_	>53.9		
	Levofloxacin (100986-85-4)	C_18_H_20_FN_3_O_4_	>54.2	6.25	1.5 × 10^7^
	Norfloxacin (70458-96-7)	C_16_H_18_FN_3_O_3_	280 (25 °C, in water)	6.34, 8.75	6.4 × 10^6^
	Ofloxacin (82419-36-1)	C_18_H_20_FN_3_O_4_	1.08 × 10^4^ (25 °C, in water)	5.97, 9.28	2.7 × 10^7^
	Sarafloxacin (98105-99-8)	C_20_H_17_F_2_N_3_O_3_	1.14 × 10^3^ (25 °C, in water)	5.6, 8.2	
Glycopeptides	Vancomycin (1404-90-6)	C_66_H_75_Cl_2_N_9_O_24_	225	2.6, 7.2, 8.6, 9.6, 10.5, 11.7	1.5 × 10^8^
Lincosamides	Clindamycin (18323-44-9)	C_18_H_33_ClN_2_O_5_S	30.61 (25 °C, in water)		131,513
	Lincomycin (154-21-2)	C_18_H_34_N_2_O_6_S	927 (25 °C, in water)	7.6	
Macrolides	Clarithromycin (81103-11-9)	C_38_H_69_NO_13_	1.693 (25 °C, in water)	8.99	7267
	Erythromycin-H_2_O (67733-56-6)	C_37_H_69_NO_14_			
	Oleandomycin (3922-90-5)	C_35_H_61_NO_12_	16 (25 °C, in water)	8.84	
	Roxithromycin (80214-83-1)	C_41_H_76_N_2_O_15_	0.0189 (25 °C)		324,384
	Spiramycin (8025-81-8)	C_43_H_74_N_2_O_14_	Slightly soluble in water	7.88, 9.28	
	Tylosin (1401-69-0)	C_46_H_77_NO_17_	5000 (25 °C, in water)	7.73	
Quinolones	Flumequine (42835-25-6)	C_14_H_12_FNO_3_	2.19 × 10^3^ (25 °C, in water)	6.5	
	Nalidixic Acid (389-08-2)	C_12_H_12_N_2_O_3_	100 (23 °C)	8.6	
	Oxolinic Acid (14698-29-4)	C_13_H_11_NO_5_			
	Pefloxacin (70458-92-3)	C_17_H_20_FN_3_O_3_	1.14 × 10^4^ (25 °C)		
	Pipemidic Acid (51940-44-4)	C_14_H_17_N_5_O_3_	322 (25 °C)		
Rifamycins	Rifamycin (6998-60-3)	C_37_H_47_NO_12_	Insoluble	1.8	
Sulfonamides	Sulfamethoxazole (723-46-6)	C_10_H_11_N_3_O_3_S	610 (37 °C, in water)	1.6, 5.7	9.8 × 10^7^
	Sulfamethazine (57-68-1)	C_12_H_14_N_4_O_2_S	1.5 × 10^3^ (29 °C, in water), 1.92 × 10^3^ (37 °C, in water)	2.07, 7.49	
	N4-Acetylsulfamethazine (100-90-3)	C_14_H_16_N_4_O_3_S			
	Sulfadimethoxine (122-11-2)	C_12_H_14_N_4_O_4_S	343		
	Sulfamethoxypyridazine (80-35-3)	C_11_H_12_N_4_O_3_S			
	Sulfapyridine (144-83-2)	C_11_H_11_N_3_O_2_S	268 (25 °C)	8.43	
	Sulfasalazine (599-79-1)	C_18_H_14_N_4_O_5_S	13 (25 °C, in water)	2.3, 6.5	
	Sulfasoxazole (127-69-5)	C_11_H_13_N_3_O_3_S	300 (37 °C, in water, pH = 4.5)	5	
	Sulfathiazole (72-14-0)	C_9_H_9_N_3_O_2_S_2_	373 (25 °C, in water)	2.2, 7.24	
Tetracyclines	Chlortetracycline (57-62-5)	C_22_H_23_ClN_2_O_8_			2.4 × 10^7^
	Doxycycline (564-25-0)	C_22_H_24_N_2_O_8_		3.5, 7.7, 9.5	8 × 10^7^
	Oxytetracycline (79-57-2)	C_22_H_24_N_2_O_9_	313 (25 °C, in water)	3.27, 9.5	9.9 × 10^9^
	Tetracycline (60-54-8)	C_22_H_24_N_2_O_8_	231 (25 °C, in water)	3.3	6.7 × 10^7^

^a^ The data come from the web of https://pubchem.ncbi.nlm.nih.gov and the literature of Gothwal et al. [18] and Fick et al. [29]. ^b^ The predicted critical effect concentration indicates that the concentration of antibiotics reaches a specific concentration after being dissolved in water, and the biological concentrate extracted from the water allows the drug concentration in fish plasma to reach a stable state equal to the concentration of a drug in human plasma.

**Table 2:** Effect of three parameters on the morphology of electrospun fiber membrane.

System parameters	Polymer molecular weight	When the molecular weight is large, the prepared fiber presents a straight line without beads and a huge diameter.
	Polymer solution concentration	An appropriate large concentration is conducive to the formation of defect-free linear fibers; excessively large concentration makes it difficult to form fibers
	Polymer solution conductivity	When the electrical conductivity increases, the fiber diameter decreases, and bead-free fiber is easily formed; excessively high electrical conductivity reduces the uniformity of the fiber diameter distribution, and normal electrospinning is not possible
	Dielectric constant	Within a specific range, the larger the dielectric constant of the same solvent, the smaller diameter of the fiber; an excessively high dielectric constant affects the jet stability
	Surface tension	Appropriate reduction of surface tension is conducive to the formation of smooth fibers
	Viscosity	Appropriately increasing the viscosity will help to form uniform bead-free fibers, but the fiber diameter will increase; extremely large viscosity results in the formation of bead-like fibers and clogging, making it challenging to perform spinning; extremely small viscosity will easily cause electrospraying
	Solvent	High solubility is conducive to the formation of fibers with good morphology; proper volatility is conducive to the spinning process and the construction of bead-free fibers
Process parameters	Applied voltage	In the proper range, the higher the voltage, the more conducive to the formation of uniform fibers with a small diameter; when the voltage is extremely high or low, the fiber diameter increases, resulting in uneven fiber diameter distribution.
	Fluid flow rate	The flow rate increases, the fiber diameter increases, and a bead-like fiber is easily formed.
	Receiving distance	Within a specific range, the smaller the receiving distance, the smaller diameter of the fiber; when the receiving distance is extremely short, the fiber diameter will increase instead.
	Diameter of spinneret	When the spinneret diameter is small, the diameter of the fiber is small.
Environmental parameters	Temperature	Within a specific range, the temperature increases, and the fiber diameter decreases.
	Humidity	Bead-like fibers are easily produced under high humidity.

**Table 3:** Preparation technology and parameters of partially electrospun functional fiber membranes for antibiotic removal.

Types of Polymers	Pretreatment Technology	Post-Treatment Technology	Effect on Antibiotic Removal	Reference
Polylactic acid	Doping TiO_2_ into PLA solution (Acetone/DMF = 3/2 *v/v*)	The fibers were deposited on fiberglass supports to prepare fiberglass fabric plain woven-type membrane, fiberglass mat-type membrane and fiberglass fabric one-fold edge-type membrane	After photocatalytic 30 min, AC was almost completely removed in aqueous solution, and the removal effect of fiberglass fabric plain woven-type membrane was the best	[12]
Polysulfone	Mixing TiO_2_/AgNPs composite particles into PSF solution (DMF/NMP = 7/3 *v/v*)	Polyamide active coating was formed on the membrane surface by interfacial polymerization	The permeability of TC resistance gene in the membrane was less than 9%	[14]
Polyethylene terephthalate	Dispersing g-C_3_N_4_ into the PET solution (HFIP)	After heat treating the PET fibers in alkaline aqueous solution in 65 °C for 1.5 h, g-C_3_N_4_ was exposed to the surface of PET and transformed from deactivation to reappearance	Under sunlight, the photocatalytic activity for the degradation of antibiotics such as SQX and SD was high, and the photodegradation rate of SQX reached 100% (pH = 5) in 2.5 h	[124]
Polyvinylpyrrolidone	Putting TiO_2_ gel into PVP solution (ethanol)	g-C_3_N_4_ nano-sheath was formed on the fiber surfaces by in-situ thermal polymerization	The photodegradation rate of TC was as high as 90.8% in 60 min, and the inactivation of *Escherichia coli* was 6 log after visible light irradiation on 90 min	[125]
Polyethylene glycol, Polyethylene terephthalate	Adding PEG into the mixed solution of g-C_3_N_4_ and PET (HFIP)	The fiber membrane was soaked in a water bath at 60 °C for 24 h, and the porous fiber was obtained by removing PEG	Under visible light, the degradation efficiency of SQX reached 100% in 2 h	[127]
Polyvinyl alcohol	Adding 20% urea to PVA solution (water)	The PVA fibers were immersed in FeCl_3_ aqueous solution for Fe^3+^ complexation and then using NaBH_4_ solution to reduce Fe^3+^, ZVI NPs was synthesized in situ and immobilized, and then freeze-dried	The adsorption equilibrium of STZ was reached in 570 min, and the electrocatalysis degradation of STZ reached 100% in 5 min	[128]

**Table 4:** Comparison of adsorption conditions and effects of different electrospun functional fiber membranes on various antibiotics.

Categories of Electrospun Functional Fiber Membranes	Types of Antibiotics	Best Adsorption Conditions	Maximum Adsorption Capacity	Reusability	Reference
PVA/SiO_2_ fiber membrane loaded with MOF	CAP	CAP concentration: 100 mg/L, Temperature: 298 K, Equilibrium time: 180 min, Oscillation rate: 200 r/min	79.5 mg/g		[13]
PAN/Fe_3_O_4_ nanofiber membrane	TC	TC concentration: 22 mg/L, Temperature: 298 K, pH = 6 ± 0.05, Equilibrium time: 72 h, Oscillation rate: 150 r/min	257.07 mg/g	Desorption agent: 0.01 M, NaOH solution, Adsorption efficiency: Only 2.72−3.61% decrease in five cycles	[130]
PI-based carbon nanofiber membrane	TC	TC concentration: 20 mg/L, Temperature: 298 K, pH = 4–7, Equilibrium time: 19 h	146.63 mg/g	Desorption agent: 1 M, NaOH solution, Adsorption efficiency: 9.3% decrease after five cycles	[131]
PAN/ZIF-8 nanofiber membrane	TC	TC concentration: 50 mg/L, Temperature: 298 K, pH = 5, Equilibrium time: 72 h	478.18 mg/g	Desorption agent: absolute ethanol, Adsorption efficiency: keeping not less than 85% of the initial adsorption capacity after five cycles	[132]
MMT/CA nanofiber membrane	CIP	CIP concentration: 10 mg/L, Temperature: 303 K, pH = 6, Equilibrium time: 60 min, Oscillation rate: 100 r/min	13.8 mg/g	Desorption agent: 10 mM NaOH solution, Adsorption efficiency: 5% decrease in CIP removal in each cycle	[149]
GO/PVDF nanofiber membrane	TC	TC concentration: 200 mg/L, Temperature: 298 K, pH = 3.8–4.2, Equilibrium time: 60 min	17.92 mg/g		[164]
PI/β-CD nanofiber membrane	TC	TC concentration: 20 mg/L, Temperature: 298 K, pH = 6, Equilibrium time: 1000 min, Oscillation rate: 120 r/min	543.48 mg/g	Desorption agent: 1 M NaOH, Adsorption efficiency: 11.51% decrease after five cycles	[165]
PAN carbon nanofiber membrane	CIP	CIP concentration: 10 mg/L, Temperature: 298 K, pH = 6.2, Equilibrium time: 8 h, Oscillation rate: 150 r/min	0.68 mmol/g		[166]
CS/PVA nanofiber membrane (crosslinked by glutaraldehyde)	TC	TC concentration: 100 mg/L, Temperature: 297 ± 2 K, pH = 6, Oscillation rate: 120 r/min	102 mg/g		[167]
Mondia white roots/PVA nanofiber membrane	Inosine, sulfamethoxazole, lidocaine, amitriptyline hydrochloride, prednisone, isoniazid, dexamethasone, ritonavir, efavirenz, fluconazole	Drug concentration: 0.5 mg/L, Temperature: 308 K, pH = 5, Balance time: 120 min, Oscillation rate: 125 r/min,	75–320 mg/g		[168]
Mesoporous protein/PVA nanofiber membrane	Sulfonamides and veterinary drugs	Sulfonamides- Drug concentration: 0.1 mg/L, Temperature: 310 K, pH = 5.5, Equilibrium time: 60 min, veterinary drugs-, Drug concentration: 0.1 mg/L, Temperature: 300 K, pH = 5.5, Equilibrium time: 60 min, Oscillation rate: 125 r/min	The removal efficiency of sulfonamides: 86.9–95.9%, The removal efficiency of veterinary drugs: 32.62–100.73 mg/g		[169]

## Data Availability

The figures referenced in this review are all licensed.

## Abbreviations

PVDF	Polyvinylidene fluoride
HFIP	1,1,1,3,3,3-hexafluoro-2-propanol
PCL	Poly caprolactone
PVP	Polyvinylpyrrolidone
DMF	*N*, *N*-dimethylformamide
MC	Methylene chloride
PI	Polyimide
NMP	*N*-methyl-2-pyrrolidone
DMAc	*N*, *N*-dimethylacetamide
DMSO	Dimethyl sulfoxide
CA	Cellulose acetate
PEO	Polyethane oxide
PS	Polystyrene
PDA	Polydopamine
PAN	Polyacrylonitrile
PES	Polyethersulfone
DTDPA	3,3′-dithiodipropionic acid
CDI	1,1-carbonyldiimidazole
AmTG	Ammonium thioglycolate aqueous solution
PET	Polyethylene terephthalate
SQX	Sulfaquinoxaline
CNCT	g-C_3_N_4_@Co-TiO_2_
PLA	Polylactic acid
AC	Ampicillin
PSF	Polysulfone
TC	Tetracycline
SD	Sulfadiazine
PEG	Polyethylene glycol
PVA	Polyvinyl alcohol
ZVI NPs	Zero-valent iron nanoparticles
STZ	Sulfathiazole
CIP	Ciprofloxacin
LEV	Levofloxacin
2,4-DCP	2,4-dichlorophenol
MB	Methylene blue
ZIF-8	Zeolitic imidazolate framework-8
MMT	Montmorillonite
CAP	Chloramphenicol
GO	Graphene oxide
β-CD	β-cyclodextrin
CS	Chitosan
SMX	Sulfamethoxazole
MTZ	Metronidazole
ERY	Erythromycin
PVB	Polyvinyl butyral
NFC	Norfloxacin
OTC	Oxytetracycline
DAS	Dialdehyde starch
GMA-S-SH	Glycidyl methacrylate-S-SH
PMMA	Poly(methyl methacrylate)
EDC	*N*-(3-dimethylaminopropyl)-*N*′-ethylcarbodiimide
NHS	*N*-hydroxysuccinimide
PLGA	Poly(lactic-co-glycolic acid)
